# Takayasu Arteritis: Criteria for Surgical Intervention Should Not Be Ignored

**DOI:** 10.1155/2013/618910

**Published:** 2013-08-06

**Authors:** A. H. Perera, J. C. Mason, J. H. Wolfe

**Affiliations:** ^1^Vascular Unit, St Mary's Hospital, Imperial College Healthcare NHS Trust, London W2 1NY, UK; ^2^Rheumatology Unit, Hammersmith Hospital, Imperial College Healthcare NHS Trust, London W12 0HS, UK

## Abstract

Takayasu aortoarteritis is a rare, chronic granulomatous panarteritis with significant morbidity amongst young patients. Current challenges include a lack of awareness about the condition, delays in diagnosis due to its varied presentation, and suboptimal methods for assessing disease activity. The development of noninvasive imaging including magnetic resonance angiography and positron emission tomography is aiding earlier diagnosis. Early initiation of immunosuppressive treatment is crucial to control active inflammation and minimize arterial injury. Recent studies investigating biological agents such as tumour necrosis factor-**α** antagonists are encouraging. Surgical revascularization should only be undertaken following careful consideration, as restenosis is common. The indications for considering intervention include uncontrolled hypertension due to renal artery stenosis, severe symptomatic coronary artery or cerebrovascular disease, severe aortic regurgitation, stenotic or occlusive lesions resulting in critical limb ischemia, and aneurysms at risk of rupture. In these cases, the risk benefit ratio for intervention is good. Open surgery, at present, has better outcomes compared to endovascular techniques. However, technological advances in endovascular treatment are continually improving. Controlling disease activity prior to and following revascularization is key to preventing complications. A multidisciplinary approach to the diagnosis and management of Takayasu arteritis is essential to achieve satisfactory patient outcomes.

## 1. Introduction

Takayasu aortoarteritis (TA) is a rare, chronic large-vessel granulomatous panarteritis of unknown aetiology, affecting the aorta and its major branches. The disease typically presents at less than 40 years of age. The aorta can be affected along its length and all branches can be involved, in addition to the pulmonary and coronary arteries. The most commonly affected branches are the subclavian artery and the common carotid artery. Histopathology reveals adventitial thickening, focal leukocytic infiltration of the tunica media, and intimal hyperplasia [1]. Arterial inflammation leads to stenotic or occlusive arterial lesions, predisposing to symptomatic end-organ ischemia. Less commonly, more acute inflammation leads to medial degeneration in the arterial wall resulting in aneurysmal dilatation [2]. Although the pattern of disease varies geographically, stenotic lesions found in >90% of patients predominate, whereas aneurysms are only reported in approximately 25% [3]. TA is associated with considerable morbidity and premature mortality amongst young patients. Poor outcomes are attributed to a delay in diagnosis, in part due to lack of awareness of the condition, late administration of medical treatment, and inappropriate patient selection and timing of vascular intervention [3]. The disease can be classified into five types on the basis of angiographic findings [4] and clinically into four groups on the basis of complications. Ishikawa defined the four most important complications of TA as retinopathy, secondary hypertension, aortic regurgitation, and aneurysm formation, each graded as mild, moderate, or severe [5]. 

## 2. Epidemiology

Rokushu Yamamoto, who practiced medicine in Japan, published the first description of Takayasu arteritis in 1830. The first scientific presentation was given in 1905 by Mikito Takayasu, Professor of ophthalmology in Japan [6]. The condition is most commonly seen in Japan, South East Asia, India, and Mexico [7]. Although early reports suggested that the disease was confined to females from Eastern Asia, it is now recognized worldwide and affects both sexes. Disease manifestations vary between regions [4], and women are affected in 80–90% of cases in the majority of cohorts. The disease commonly presents in the second or third decade of life, with a delay in diagnosis of between months and years from the onset of first symptoms. However, the illness may also present in childhood [8]. The mean delay in diagnosis is significant and varies from 10 months to 4.9 years, predisposing to severe arterial injury [8, 9]. In a striking recent survey, ninety-one percent of patients reported seeing more than one physician prior to diagnosis (mean 5.1, median 4 physicians) [10]. The incidence of TA appears to vary in different geographical areas: it was calculated to be 1-2/million in Japan and 0.8/million in Sweden and in the UK [11–13]. 

## 3. Signs and Symptoms

Clinical signs and symptoms arise from both systemic inflammation and local vascular complications. The clinical manifestations usually follow two phases [14]. In the early phase patients may complain of systemic symptoms including fever, weight loss, malaise, myalgia, carotidynia, and headaches. These symptoms are often insidious and frequently missed, or their significance is overlooked. The later pulseless phase, commonly appearing months or years later, reflects end-organ ischemia [8]. Focal manifestations differ according to the anatomical location of the affected arteries. Symptoms of upper and lower limbs claudication, light headedness, and chest pain predominate. Decreased or absent peripheral pulses, aortic regurgitation, arterial bruits, and both systemic and pulmonary hypertension are common. The most common disease manifestations are listed in Table 1 [8, 15].

## 4. Morbidity and Mortality

Morbidity is significant, with 23% of patients unable to work and 60% experiencing limitations in their activities of daily living [15]. A study of quality of life (QOL) and physical and mental health scores in 158 patients with TA revealed that the disease has profound consequences on QOL, with poorer physical and mental health scores compared to other chronic diseases [10]. Remission of the disease was a significant predictor of improvement in both physical QOL (*P* = 0.0002) and mental QOL (*P* = 0.002). 

Mortality rates vary based on geographical location and are likely due to variations in management strategy. They vary from 3% in the United States in 2 studies from 1994 and 2006 to 11% in India from a study in 1996 [8, 15, 16]. A Japanese study demonstrates improvements in 15-year survival rates from 79.9% for patients diagnosed between 1957 and 1975 to 96.5% in those diagnosed between 1976 and 1990 [9]. This is likely to reflect improved knowledge of the condition, advances in imaging, earlier diagnosis, and tailored immunosuppression and surgical treatment. Main causes of death include congestive cardiac failure, acute myocardial infarction, stroke, and postoperative complications [9]. 

## 5. Current Challenges

There remains a lack of awareness about the condition [3]. This is compounded by the spectrum of disease presentation and nonspecific symptoms, which provide further challenges in making a definitive diagnosis. The low prevalence of the condition makes it difficult to provide a standardised approach for diagnosis and treatment, particularly in the West. Once diagnosed, a key limitation to optimal management is the relative lack of sensitive and accurate means for monitoring disease activity.

## 6. Diagnosis and Assessment of Disease Activity

The spectrum of presentation, disease severity, and pace of progression can often lead to inaccurate assessment and delay in diagnosis [8]. Diagnosis is mainly based on physician awareness, along with a high index of suspicion. If TA is suspected, it is essential to palpate peripheral pulses, listen for bruits, and measure blood pressure in all four limbs. Blood tests should be evaluated for evidence of an acute phase response (elevated Erythrocyte Sedimentation Rate [ESR] and C-Reactive Protein [CRP]) and normochromic normocytic anaemia [14], and the diagnosis is confirmed on imaging. Patients present to a variety of medical specialities, and management can be scattered and often suboptimal as a result. Therefore it is imperative that all specialities, especially primary care doctors, cardiologists, ophthalmologists, and cardiac and vascular surgeons, in addition to rheumatologists, understand the natural history of the diseases and the options for medical and surgical intervention.

Takayasu aortoarteritis has a diverse disease course but most commonly consists of active and quiescent phases. Assessment of disease activity is therefore key but can be challenging. The lack of biopsy material further compounds this situation. Ideally the diagnosis should be made early in the prestenotic phase which would permit initiation of treatment to suppress inflammation and prevent vascular injury [3]. There are however limitations in the diagnostic criteria. The American College of Rheumatology and Ishikawa criteria favour the detection of established stenotic disease [17, 18]. Incorporation of the recently developed Indian Takayasu Activity Score (ITAS) will improve assessment of disease activity [19]. ITAS has been developed in light of the relative insensitivity of the Birmingham Vasculitis Activity Score (BVAS) in patients with TA [20]. ITAS includes an expanded cardiovascular component reflecting active disease, with an emphasis on bruits, pulse loss, and claudication. Serial assessments show that ITAS also reflects response to medical treatment and detects flare-ups. For patients with TA, periodic imaging with magnetic resonance angiography assists in the assessment of arterial anatomy and disease progress, but it is not a reliable indicator of disease activity [21]. All patients should remain under long-term surveillance. 

## 7. Serological Markers

Measurements of ESR and CRP are useful diagnostic adjuncts. However there are no specific autoantibodies or serological disease biomarkers that can reliably distinguish between healthy volunteers and active TA [3, 22]. Furthermore, Cong et al. reported biopsy data that revealed active inflammation in 58.3% of patients thought to be in clinical remission, all of whom had a normal ESR [23]. Thus it is essential that one does not rely on acute phase reactants alone but uses a combination of clinical examination, assessment of patient symptoms, and imaging studies to exclude progressive arterial injury [3]. Notwithstanding, it has been shown that vascular complications are more likely to occur when biological inflammation (raised ESR and serum levels of CRP and fibrinogen) is present at the time of the vascular revascularization, regardless of the modality of treatment [24]. Therefore monitoring of these markers and escalation of immunosuppression, particularly prior to embarking upon surgical intervention, are key. However, novel disease biomarkers are required. Two recent cross-sectional studies have suggested a potential role for pentraxin-3 (PTX-3) [25, 26]. This prototype long pentraxin is involved in innate immunity, acts as an acute phase reactant, and has been identified in the arterial wall in TA. The plasma levels of PTX-3 had greater accuracy than ESR and CRP for distinguishing active from inactive disease and were not influenced by steroid therapy. Further prospective studies are required to determine whether PTX-3 levels will be useful in the care of patients with TA in clinical practice.

## 8. Imaging

Percutaneous digital subtraction radiographic angiography was previously considered the gold standard for diagnosis of TA, but recent advances in noninvasive vascular imaging have provided new insights into TA [17]. In addition, angiography has limited value in early detection of vascular lesions prior to luminal changes. Its invasive nature, contrast-requirement, and high radiation dose limit its use in long-term follow-up. 

**Figure 1:**
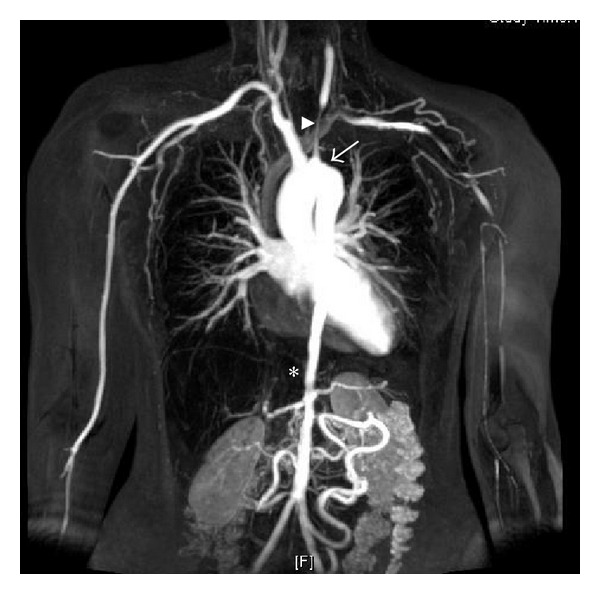
Magnetic resonance angiography in Takayasu arteritis. Contrast-enhanced magnetic resonance angiogram revealing the extent of arterial disease in a young woman with Takayasu arteritis. The left subclavian artery is occluded (arrow) and this has led to collateral formation. There is a long stenosis in the left common carotid artery (arrowhead). The lower thoracic and abdominal aorta is narrow and irregular down to the aortic bifurcation (star).

Contrast-enhanced computed tomography angiography (CTA) and particularly magnetic resonance angiography (MRA) (Figure 1) can demonstrate arterial anatomy, wall enhancement, oedema, and thickening which might enable early disease detection where luminal diameter is still preserved [3]. 

**Figure 2:**
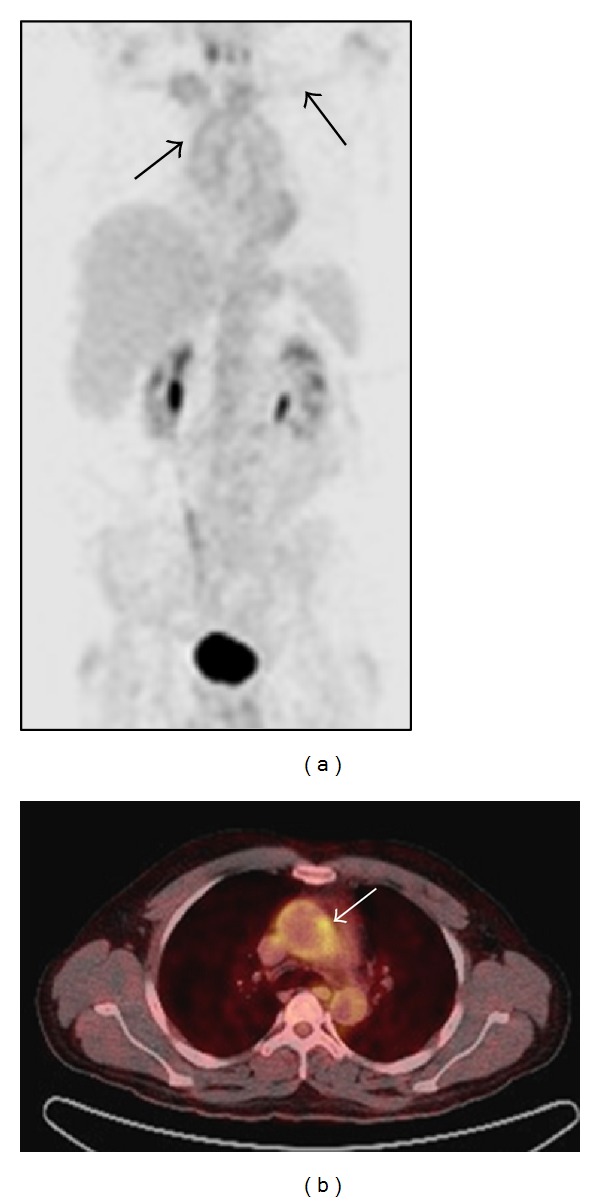
Fluorodeoxyglucose positron emission tomography. (a) An ^18^F-FDG-PET scan in a young woman with active Takayasu arteritis demonstrating homogeneous linear uptake of FDG in the arch of the aorta and the subclavian arteries, suggestive of active disease. (b) Coregistered ^18^F-FDG-PET-CT image demonstrating increased uptake localised to the wall of the thoracic aorta of a man of 26 years with active disease.

Positron emission tomography with ^18^F-FDG (^18^F-FDG-PET) is a functional imaging modality that highlights areas of increased metabolic activity and is thereby useful to detect inflammation with a reported high sensitivity and specificity in TA [27]. Increasingly, combined ^18^F-FDG-PET-CT scanners are being used with the coregistered images allowing more precise anatomical information of metabolic activity and hence an increased sensitivity, allowing diagnosis of early prestenotic disease [28]. In active disease a homogeneous linear pattern of ligand uptake is typically seen in affected arteries and most commonly in the aorta, subclavian and common carotid arteries (Figure 2). However, there are limitations with this technique, which include a lack of standardised technique for quantification of uptake, limited availability, and lack of reliable evidence for evaluation of disease activity [29]. 

High-resolution colour duplex ultrasound (US) is utilised in the assessment of common carotid and proximal subclavian arteries in TA and can also be used to detect and monitor abdominal aortic aneurysms [30]. The typical lesion identified by US in TA is a long, smooth, homogeneous concentric thickening of the arterial wall. This is in contrast to atherosclerotic plaque, which is nonhomogeneous, irregular, and often calcified. Duplex US also has the potential to be used as an index of disease activity based on measurements of wall thickness [31]. It is however a technique that is operator dependent and has defined and limited use. 

For the diagnosis of early prestenotic disease ^18^F-FDG-PET-CT is the modality of choice. Diagnosis following a more typical presentation in the stenosis phase can be made with MRA or CTA, with PET scanning reserved for assessing disease activity [3]. Percutaneous angiography is of value when planning vascular intervention, and MRA is favourable for long-term monitoring of disease progression. 

## 9. Medical Treatment

The aim of medical treatment is to control active inflammation and minimize arterial injury. To prevent the development of vascular complications and induce remission, early initiation of immunosuppressive treatment is crucial [15, 32]. Prednisolone is the first line agent, and the EULAR (European League Against Rheumatism) guidelines recommend an initial dose of 1 mg/kg/day (total maximum dose 60 mg/day), with gradual tapering [32]. Adjunctive steroid-sparing immunosuppression is required in the majority of patients to minimise steroid-related complications and control disease progression, particularly as there is considerable risk of relapse when steroid treatment is stopped [15]. There are studies suggesting methotrexate and azathioprine are effective at inducing remission and halting progress of arterial lesions [33, 34]. Mycophenolate mofetil has also been shown to be safe and effective as a steroid-sparing agent in a small open-label study [35]. Pulsed cyclophosphamide has been reported as effective, particularly in cases resistant to glucocorticosteroids, though its use should be restricted to extremely severe cases due to its association with infertility and increased toxicity [36]. There is emerging literature in support of the use of biological agents in the treatment of TA. A recent review published of 84 patients with TA treated with tumour necrosis factor-*α* antagonists including infliximab and etanercept revealed complete remission in 37%, partial remission in 53.5%, and 9.5% nonresponders [37]. Of note, side effects (mainly infections and hypersensitivity reactions) were observed in 20% of cases. Likewise, anti-IL-6 receptor monoclonal antibody tocilizumab may help control refractory disease and the published cases have recently been reviewed in the literature [38]. It is also imperative that cardiovascular risk factors, accelerated atherosclerosis, and systemic and pulmonary hypertension are investigated and treated actively where appropriate [3]. 

## 10. Surgical Treatment

### 10.1. Indications for Intervention

**Figure 3:**
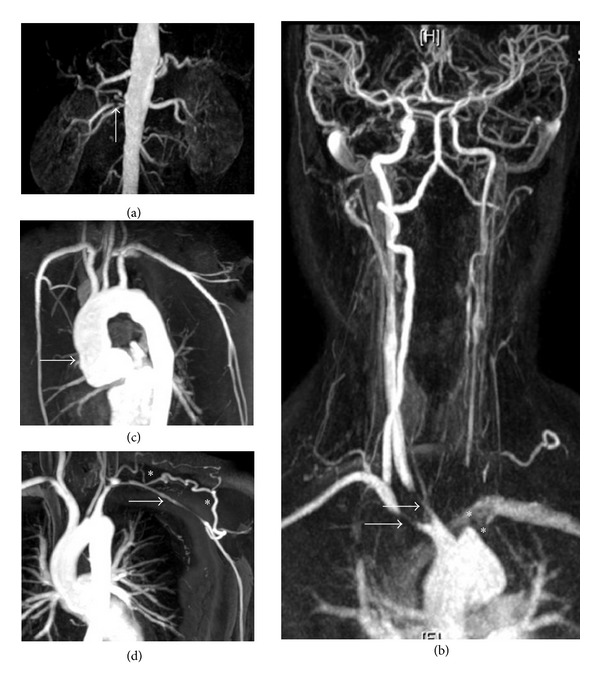
Indications for surgical intervention in Takayasu arteritis. Surgery should not be undertaken lightly in patients with TA. However, the outcome of intervention in those with serious complications is good and improved if disease activity can be controlled prior to surgery. The figure shows MRA studies. (a) A right renal artery stenosis subsequently successfully treated by percutaneous angioplasty with resolution of hypertension. (b) Severe disease of the great vessels including stenoses of the right subclavian and common carotid arteries (arrows) and occlusion of the left subclavian and left common carotid arteries (stars). The patient suffered severe cerebrovascular symptoms and underwent successful bypass surgery. (c) A dilated ascending aorta with associated severe aortic regurgitation in a young female patient who required aortic valve replacement. (d) Although patients commonly suffer claudication symptoms as a consequence of subclavian artery stenosis (arrow), these often improve following the development of collaterals (stars) as shown here, precluding the need for intervention.

With symptomatic stenotic or occlusive lesions, it appears appropriate and often necessary to revascularize. The published literature however indicates that these procedures should be carefully considered and that restenosis is common; therefore intervention should be reserved for specific indications (Figure 3) [3, 8, 15, 23]. Rates of surgical intervention vary between centres from 12% to 70%, but it seems likely that less than 20% of patients with TA actually require revascularization [9, 15, 39]. The indications for considering intervention include uncontrolled hypertension as a consequence of renal artery stenosis, severe symptomatic coronary artery or cerebrovascular disease, severe aortic regurgitation or coarctation, stenotic or occlusive lesions resulting in critical limb ischemia, and aneurysms at risk of rupture. In these cases the risk benefit ratio for surgery is good [9, 39, 40]. Miyata et al. demonstrated that surgery increases the long-term survival of patients with stage 3 TA (major complication and progressive disease, based on prognostic classification by Ishikawa and Maetani [9]), while conversely survival is decreased in stage 1 patients (no major complications and no evidence of progressive disease) due to surgery-related complications [40]. Therefore conservative medical management is recommended for patients with stage 1 and 2 disease. 

### 10.2. Complications

 Long-term survival rates following surgical bypass procedures are good [24, 39, 40]. Miyata et al. demonstrated a cumulative incidence of anastomotic aneurysms following surgery of 13.8% at 20 years [40]. Therefore lifelong regular follow-up is mandatory for these patients. The results for endovascular intervention (angioplasty and stenting) are less encouraging in comparison [23, 24]. This might in part reflect the character of the TA lesions [3]. Takayasu lesions in aortic branch vessels are frequently proximal and can render placement of a stent difficult. The lesions are often long, irregular, and fibrosed, thereby predisposing to restenosis. Good results are seen in the treatment of short focal stenosis in the presence of inactive disease [41, 42]. Surgical treatment had a better outcome compared to endovascular intervention in terms of postoperative complications (37.5% and 50%, resp.) [24]. Cong et al. echo this, where restenosis occurred in 34.7% of surgical bypasses and 77.3% of angioplasty procedures [23]. The main complications of all intervention, both open surgery and endovascular techniques, include restenosis (75.7%), thrombosis (10%), bleeding (8.6%), and stroke (5.7%) [24]. 

### 10.3. Renal Artery Lesions

Ham et al. reported outcomes of 79 renal artery interventions (62 renal bypasses, 12 endovascular interventions [8 angioplasty and 4 stents], and 5 nephrectomies) on a cohort of patients with nonatherosclerotic renal artery disease (NARAD), of which more than 55% had TA [43]. While both methods conferred long-term benefits, open revascularization demonstrated superior 1-year and 5-year outcomes including patency rates (91% and 80%, resp.) compared to endovascular intervention (73% and 49%). They concluded that open revascularization should be considered selectively as first line therapy for NARAD, particularly in young patients with moderate and complex renal artery disease. Feng et al. also concluded that aortorenal bypass in TA-induced renal artery stenosis is safe, effective, and durable at 5-year follow-up (79% primary patency rate) [44]. However, in the renal arteries, where lesions are often proximal and short, good results can be obtained with percutaneous angioplasty. Sharma and Gupta treated 264 renal arteries in 193 patients with renovascular hypertension secondary to TA [41]. Clinical benefit was seen in 91% of patients, with improvement in hypertension, decreased need for antihypertensive medication, and a cumulative 5-year patency rate of 67%. Restenosis was seen in 17% of lesions, all of which were successfully retreated. The group concluded that renal angioplasty is more suitable than primary stenting in this young cohort, firstly due to technical difficulties such as small access vessels, frequent vessel spasm during manipulation, and coexisting perirenal aortic stenosis and as the long length of the stenosis often provides insufficient landing zones for stenting. In addition, the tough nature of these stenoses can lead to incomplete stent expansion and there is an increased risk of in-stent restenosis following stenting. Lastly, renal artery stents complicate any subsequent surgical revascularization. Tyagi et al. performed percutaneous angioplasty on 54 TA patients with hypertension and renal artery stenosis [42]. They showed a 93% improvement in blood pressure. Restenosis occurred in 13.5%, all successfully dilated. Despite these encouraging results, the restenosis rate with endovascular intervention in TA is variable and significantly higher than that seen with atherosclerotic disease [45]. Qureshi et al. suggest improved outcomes with the use of covered stent grafts, where they showed improved patency and fewer secondary interventions in comparison to bare metal stents [46]. This approach merits further investigation.

### 10.4. Carotid and Subclavian Artery Lesions

 Common carotid and subclavian artery lesions often have long, irregularly fibrosed stenotic segments, seldom amenable to angioplasty or stenting. Kim et al. demonstrated superior patency of supra-aortic vessels with surgical bypass (12.5% restenosis) compared to endovascular intervention (53.3% restenosis), despite the use of endovascular intervention for short stenotic lesions (<5 cm) and bypass for longer occlusive lesions [47]. However, more serious postoperative complications occurred in the bypass group. It is vital that revascularization is not performed on the basis of radiological stenosis or occlusion, but in combination with symptom assessment to determine the need for intervention. This is illustrated by the fact that upper limb claudication secondary to subclavian artery stenosis often improves following the development of collateral circulation and seldom requires intervention [3]. Cerebrovascular and upper limb angiography can be alarming, but frequently the young patients remain asymptomatic and can fall into stage 1 or 2 disease where revascularization is not required.

### 10.5. Coronary Artery Lesions

 Endo et al. found coronary artery stenosis or occlusion to be the most common cardiac abnormality associated with TA, with a lower incidence of aneurysmal coronary ectasia and coronary steal phenomenon [48]. They recommend treatment of coronary stenosis, as coronary ischemia is one of the main causes of death. They demonstrated satisfactory outcome following surgical treatment of coronary artery disease with an actuarial survival rate, including in-hospital deaths, of 86.5% at 5 years. Matsuura et al. demonstrated a favourable late outcome of surgical treatment (aortic valve replacement or composite graft repair) of aortic regurgitation due to TA in 90 patients, with an overall 15-year survival rate of 76.1% [49]. Importantly, univariate analysis revealed active inflammation to be a risk factor for detachment of the valve or graft, emphasizing the necessity of preoperative and postoperative immunosuppression where possible. 

### 10.6. Success of Intervention

There have been several studies highlighting the importance of achieving disease remission prior to revascularization [24, 39, 50]. Fields et al. showed patients with active disease undergoing revascularization are more likely to require revision or develop progressive symptomatic disease at another site [39]. Saadoun et al. demonstrated by multivariate analysis that biological inflammation at the time of revascularization was independently associated with the occurrence of arterial complications after a vascular procedure [24]. According to the literature, open surgical treatment remains superior to endovascular intervention in the treatment of TA lesions. However, the recent results of endovascular procedures are encouraging, and we await new technological advances. Correlation of imaging with symptoms, adherence to the indications for revascularization in TA, and ensuring disease activity is controlled prior to and following a procedure are key to the success of any vascular intervention, regardless of modality. 

## 11. Prognosis

The overall prognosis of TA has improved over the past decade. This is most likely due to improved earlier diagnosis secondary to the development of noninvasive diagnostic imaging [51]. In a study of 106 TA patients at a Japanese institution, the time from onset of symptoms to diagnosis was significantly shortened (*P* = 0.0005) during the decade, with a delay in diagnosis of 5.2 years before 1999 and 1.2 years after 2000 [51]. Combined use of imaging tools to improve the diagnosis has increased and the frequency of occlusion of branches of the aortic arch and incidence of moderate/severe aortic regurgitation has significantly decreased. The number of patients treated with immunosuppressive agents has also increased significantly. Overall, surgical outcomes have improved over time, aided by advances in surgical techniques and technology as well as improvements in preoperative optimisation and peri- and postoperative care. Due to the varied presentation and course of the condition in addition to the existing challenges, a multidisciplinary approach to the diagnosis and management of Takayasu arteritis patients is essential to achieve satisfactory patient outcomes.

## Figures and Tables

**Table 1:** Common disease manifestations.

(i) Diminished or absent pulses
(ii) Blood pressure discrepancy between the arms (>10 mmHg)
(iii) Fatigue
(iv) Arterial bruit
(v) Headache
(vi) Limb claudication
(vii) Fever
(viii) Light headedness
(ix) Hypertension
(x) Arthalgia/myalgia
(xi) Carotidynia
(xii) Stroke
(xiii) Angina
(xiv) Aortic regurgitation
(xv) Weight loss
(xvi) Anaemia
